# Variable Strength of Forest Stand Attributes and Weather Conditions on the Questing Activity of *Ixodes ricinus* Ticks over Years in Managed Forests

**DOI:** 10.1371/journal.pone.0055365

**Published:** 2013-01-25

**Authors:** Ralf Lauterbach, Konstans Wells, Robert B. O'Hara, Elisabeth K. V. Kalko, Swen C. Renner

**Affiliations:** 1 Institute of Experimental Ecology, University of Ulm, Ulm, Germany; 2 Biodiversity and Climate Research Centre, Frankfurt, Germany; 3 Smithsonian Conservation Biology Institute, National Zoological Park, Front Royal, United States of America; USDA-Agricultural Research Service, United States of America

## Abstract

Given the ever-increasing human impact through land use and climate change on the environment, we crucially need to achieve a better understanding of those factors that influence the questing activity of ixodid ticks, a major disease-transmitting vector in temperate forests. We investigated variation in the relative questing nymph densities of *Ixodes ricinus* in differently managed forest types for three years (2008–2010) in SW Germany by drag sampling. We used a hierarchical Bayesian modeling approach to examine the relative effects of habitat and weather and to consider possible nested structures of habitat and climate forces. The questing activity of nymphs was considerably larger in young forest successional stages of thicket compared with pole wood and timber stages. Questing nymph density increased markedly with milder winter temperatures. Generally, the relative strength of the various environmental forces on questing nymph density differed across years. In particular, winter temperature had a negative effect on tick activity across sites in 2008 in contrast to the overall effect of temperature across years. Our results suggest that forest management practices have important impacts on questing nymph density. Variable weather conditions, however, might override the effects of forest management practices on the fluctuations and dynamics of tick populations and activity over years, in particular, the preceding winter temperatures. Therefore, robust predictions and the detection of possible interactions and nested structures of habitat and climate forces can only be quantified through the collection of long-term data. Such data are particularly important with regard to future scenarios of forest management and climate warming.

## Introduction

If we are to understand variation in occurrence patterns and population dynamics, and how these are affected by global change we need to quantify the important environmental forces so that we can improve our predictions and management decisions. The integration of organisms in their environment involves complex interactions with abiotic and biotic factors. For instance, within a network of host-parasite species, the various taxa not only interact with each other, but are also concomitantly affected by the underlying habitat and climate conditions [1], [2]. Moreover, variation in environmental conditions has been shown to be a major driver of population fluctuations [3]–[6]. Although time series analyses of long-term data are a valuable tool for disentangling possible drivers of population fluctuations and spatiotemporal heterogeneity, many studies of particular organisms lack data collected over several years. Hence, studies of the way that focal organism respond to environmental gradients frequently rely on single years or on data sets pooled over a limited number of consecutive sampling years. Generalizations about the response of species to various habitat conditions are also challenged by the inconsistent relative impact of environmental forces over years [7], [8]. It is therefore critical to ask how those forces may change over years, and what kind of conclusions can be confidentially drawn from short-term studies.

Ixodid ticks are a major pathogen-transmitting vector in temperate forests. Ticks are ectotherms that spend the largest proportion of their life off-host. They ambush their vertebrate host in vegetation and can survive adverse winter conditions in leaf litter [9]. Abiotic factors, such as temperature, humidity, and elevation, are key factors that determine the development and survival of ticks [10]–[13]. In particular, microclimatic conditions play an important role in the questing and feeding behavior in ticks, such that questing activity in unfed nymphs decreases in upper vegetation layers with increasingly dry conditions [14].

Another major factor affecting tick occurrence and density is their access to vertebrate host species [15]. Both abiotic conditions and host access, as major sources of variation in tick occurrence and density, interplay in a complex manner with local habitat and vegetation conditions. At the local scale of diverse forest types, vegetation structure has profound effects on the microclimate relevant to ticks [16], [17]. Vegetation structures, such as the near-ground vegetation, litter, and the upper soil, are, in turn, largely determined nowadays by forest management practices [18]. In managed and modified temperate production forests, forest stand characteristics such as tree composition, stratification, and the intensity of silvicultural practices are key drivers of species occurrences [19].

Together with habitat conditions, climate (in particular temperature and precipitation) are expected to change considerably during the next few years and decades as part of ongoing global change [20], [21]. At a regional scale, climate conditions such as warmer winters and drier spring periods are likely to alter tick survival and distribution [22], [23]. On the local scale, tick survival and density can be expected to be mainly affected by the overall regional climate, together with local microclimatic conditions, which are, in turn, driven by local habitat features and topography [24]. Ticks have been found to be less sensitive to drought in deciduous than in coniferous temperate forests [17], [25]. Given that managed forest continue to change at the stand level through silvicultural practices [26], and that the management practices induce compositional changes in the vegetation toward drought-tolerant species [27], an understanding of those environmental factors that influence tick occurrence and associated disease risk is important.

In this study, our aim has been to analyze observations from a monitoring program to increase our understanding of the way that climate and habitat conditions have impacted tick density in differently managed forest stands in SW Germany since 2008. One of our main interests has been to investigate the aspect of forest management that most importantly influences questing nymph densities: (1) tree composition, (2) stand type, or (3) forest successional stage. The last-mentioned has rarely been considered to date but comprises a forest classification that is commonly used by forest management authorities. Forest successional stage classification might therefore aid in regional tick and disease risk mapping. We have further assessed whether the relative impact of environmental forces on tick density differs over years and discuss the shortcoming of studies with limited spatiotemporal replicates.

## Materials and Methods

### Study site and data collection

As part of the interdisciplinary long-term project ‘Biodiversity Exploratories’ (www.biodiversity-exploratories.de), 50 permanently marked experimental plots in different forest stands in the Schwabian Alb, SW Germany (48°25′ N, 9°23′ E) have been established. All plots comprise relatively homogeneous forest cover over 100×100 m [28]. This region comprises a submontane Karst area (elevation 500–900 m a.s.l.) with annual average temperatures between 6 and 7°C and a vegetation period of about 200 days [28]. The land cover was originally dominated by the common beech (*Fagus sylvatica*) but has substantially changed during the long history of human land use involving the clearance of parts of the forests and various farming practices resulting in a mosaic landscape covered with about 41% forest, 24% grassland, and 22% fields (unpublished data). Forest stands range from the remains of old-growth beech forest, through continuous stands with low-intensive logging, to actively managed deciduous, mixed deciduous-coniferous, and coniferous age-class forests. In addition to beech, common ash (*Fraxinus excelsior*) and sycamore (*Acer pseudoplatanus*) are the most common deciduous tree species in this area. Norway spruce (*Picea abies*) monocultures have been largely planted and comprise about 20% of forest stands.

Questing ticks were sampled in the summer (June to August) between 2008 and 2010 by dragging a white woolen cotton cloth of 80×80 cm in size. The cloth was sewn at one end to a thin metal pole for support and spread over the forest floor and lower ground vegetation. We sampled ticks in a sampling area of 12×12 m in 49 out of 50 of the experimental plots. The same areas were sampled in consecutive years. The site of the sampling areas was chosen next to permanently installed game-exclusion sites (fenced 12×12 m). We systematically avoided areas with dense vegetation growth or large numbers of saplings or piles of dead wood, as the efficiency of drag sampling is largely biased by vegetation height [29]. For sampling, the cloth was systematically dragged over the sampling area, being held as close to the ground as possible with the aid of a telescope rod. We sampled all plots in the same fashion regardless of vegetation type and ground cover. In order to ensure equivalent sampling of all plots, dragging within sampling areas was performed in six intervals of ca. 50 meters (covering different parts of the sampling area) after which we examined the cloth and used fine forceps to remove all ticks. These intervals are relatively large and might not be appropriate to capture as many ticks as possible compared with intervals of only 5 meters [29]. While we acknowledge this shortcoming, we expect the same bias to occur on all plots and thus consider that our sampling provides a meaningful measure of relative tick questing activity. Ticks were sampled during the daytime (08∶00 to 17∶00) on days without rain. Because of the large-scale design of the research platform and workload, we were not able to sample all plots simultaneously. Different plots were sampled during a period of 53 days in the initial year and of 15 days in the second and third years.

Tick activity changes rapidly throughout the season, and sampling over a longer time period may thus bias density estimates. We found, however, in a preliminary analysis of correcting for sampling time as a random effect in the analysis (outlined below) only a negligible effect of sampling time on the number of ticks collected under various environmental conditions; we thus present our results without this random effect.

Ticks were preserved in 80% ethanol for later identification and counting. For habitat classification, we categorized forest stand composition (based on beech and spruce as the dominant overstory tree species, with a threshold level of 70% of dominant trees) into three categories: beech, beech-mixed, and spruce. We further distinguished successional stages (thicket, pole wood, young timber, old timber; see [28]). We estimated herb coverage of the sampling area as the percentage of area covered with any herbaceous plant. We estimated shrub coverage as the coverage of sampling area with all shrubs and tree saplings between 30 and 200 cm in height. Soils were classified as cambisols or leptosols, based on extensive soil profile examination and were included as a covariate. We expected soil attributes to contribute to microclimatic conditions (I. Schöning, Uni Jena; see [28]).

We used gridded weather data from the German Weather Service (*Deutscher Wetterdienst*, [30]), which interpolates daily measured data from weather stations to a spatial grid with 1 km^2^ resolution as monthly means. We calculated averaged temperature and averaged precipitation in the winter (December to January) and spring (March to May) from these monthly data preceding our sampling efforts. Here, we assumed precipitation as an indicator of relative humidity or water saturation deficit, which is known to be an important driver of questing behavior in *I. ricinus* [31].

As the precipitation and temperature in the spring was highly positively correlated, we used only winter temperature and winter and spring precipitation in our analysis. We calculated yearly averaged weather conditions as the yearly means of z-scores of weather data.

For quantitative analysis of tick questing activity in relation to environmental covariates, we considered only nymphs, as larvae are commonly spatially clustered.

All data are deposited in the Biodiversity Exploratories Information System (BExIS; http://www.biodiversity-exploratories.de/) and are available upon request from the authors according to project policy.

### Data analysis

Hierarchical and multilevel modeling approaches allow inference to be made with regard to group-specific parameters, whilst also estimating the variation between the groups. This allows decomposition of the variation among potential covariates and indicates those that are important for explaining the overall variation [32], [33]. With such models, one can explicitly focus on the variance in both the spatial and temporal dimension. In addition, uncertainty in predicting effects of covariates that might themselves vary in relative effects among spatiotemporal dimensions are adequately considered in such models.

We employed a generalized linear mixed model based on Bayesian Markov-Chain Monte Carlo methods to estimate the relative effects of environmental covariates on the spatiotemporal variation in questing nymph density. We used the software package MCMCglmm [34] in R [35]. Within this framework, some parameters describing the corresponding effects of explanatory variables were treated as random variables with assumed hyper-prior distribution, and so posterior distribution of parameters were estimable together with the possibility of specifying the (co)variance of the random effects.

We assumed a generalized linear regression model for investigating the link between the variability in questing nymph density and the underlying environmental covariates.

In a first step, the density of questing nymphs at various sites in the different years can be described as:

Number of nymphs ∼ (1|Sites) + Forest Type + Successional Stage + Herb Coverage + Shrub Coverage + Soil + Elevation + Winter Temperature, Winter Precipitation + Spring Precipitation.

where (1|Sites) corresponds to a variable intercept (‘mean tick number’) which is allowed to vary over sites fitted as random effects. In addition, constant coefficients are estimated for environmental variables. Random noise is added from an assumed Poisson distribution (note that the MCMC approach accounts for the overdispersion in data).

The hierarchical model structure can be most easily explained with the variable intercept α*_Sites_* assumption: the level of allowing the mean to vary across sites from a common mean μ_α_ is modeled from a normal distribution with the respective variance σ_α_^2^ by α*_Sites_* ∼ N(μ_α_, σ_α_^2^).

The size of σ_α_^2^ allows the identification of the divergence of site-specific means from the common mean, i.e., σ_α_^2^ = 0 leads to complete pooling across sites, while σ_α_^2^>>0 suggests that mean tick densities vary greatly across sites, resulting in different posterior distributions for site-specific estimates [33].

As a preliminary characterization of whether overall yearly averaged winter temperature (in a colder winter, for example, temperatures generally dropped at all sites, and our data included more variation among years than within years in weather covariates) have an impact on the relative effects of forest type and stage, we fitted additional random effects by including yearly averaged winter temperature into the intercept and allowing them to vary by forest type and stage:

Number of nymphs ∼ (1|Sites) + Forest Type + Successional Stage + Herb Coverage + Shrub Coverage + Soil + Elevation + Winter Temperature + Winter Precipitation + Spring Precipitation + (Yearly Averaged Winter Temperature| Forest Type + Successional Stage).

In principal, in such a random regression framework, an increase in the variance for the added random effects means that the identity of the different habitat types might explain the larger amounts of variation depending on yearly weather conditions.

We also fitted models for single year datasets with removed random effects.

We standardized all numerical explanatory variables so that they each had a mean of zero and standard deviation of one. Posteriors were gathered by running 350,000 iterations, discarding 100,000 iterations, and thinning by 25, resulting in 10,000 draws for posterior estimates. Convergence and mixing was checked visually by running two parallel chains. Results are given as the posterior mode and 95% highest posterior density as the credible interval (CI).

## Results

During the three years of sampling, we collected a total of 3,355 larvae and 855 nymphs of the species *Ixodes ricinus*.

**Figure 1:**
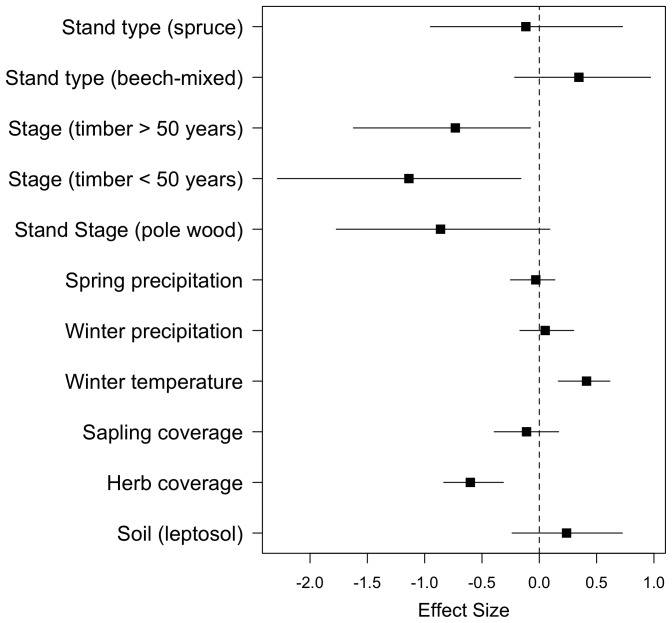
Effect sizes of environmental covariates on questing nymph densities from the full Bayesian MCMC generalized linear model comprising three years of sampling (2008–2010). Effect sizes are given as posterior modes and 95% highest posterior density estimates. Note: the baseline for stand type is ‘beech’ and for stand stage ‘thicket’.

In the full models for nymphs (analysis of larvae is not considered because of their clustered occurrence), the posterior densities revealed significant effects of habitat features with fewer nymphs being sampled in older forests stands of timber and pole wood compared with the earlier stage of thicket (Figure 1), which accounted for 17% of the variation in abundances (nymphs). Questing nymph density significantly decreased with increasing herb coverage (explaining 25% of variance).

**Figure 2:**
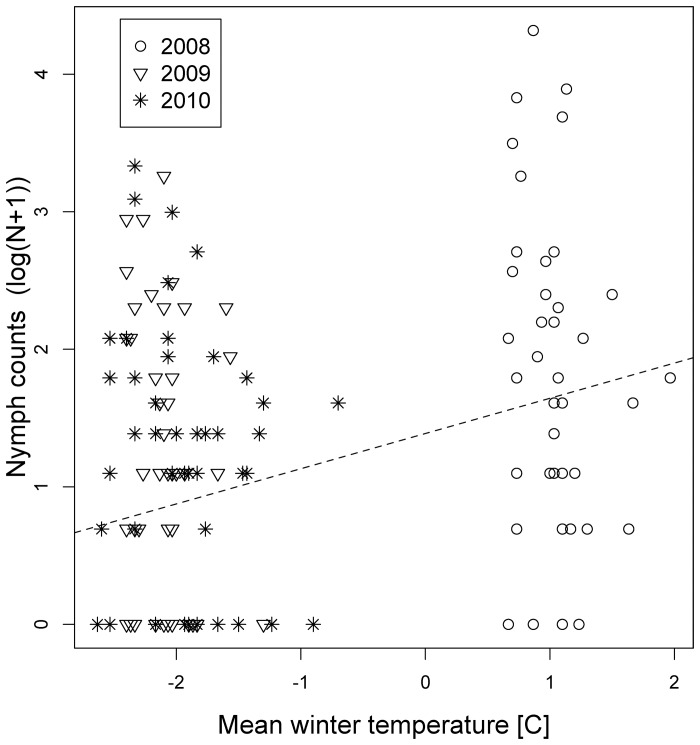
Relationships between the number of counted tick nymphs and mean winter temperatures from 2008–2010. The Y-axis comprises log-transformed (log(counts+1)) count data. The winter in 2008 was considerably warmer than the two following years. Hence, the respective values cluster on the right site of plots. Simple regression lines from the linear model relationships show the trend of increasing nymph densities with milder winter temperatures.

In the full models for nymphs, winter temperature was associated with an increase in density, with a change of 1°C increasing relative questing nymph density by 82% (Figure 2) and explaining 12% of the variance.

The random effect of yearly averaged weather conditions revealed no considerable effects on habitat identity effects, and we were hence not able to show possible interaction effects between habitat and weather. Since the posterior distributions largely resembled prior distributions, this was most likely attributable to the small sample size of three years.

**Figure 3:**
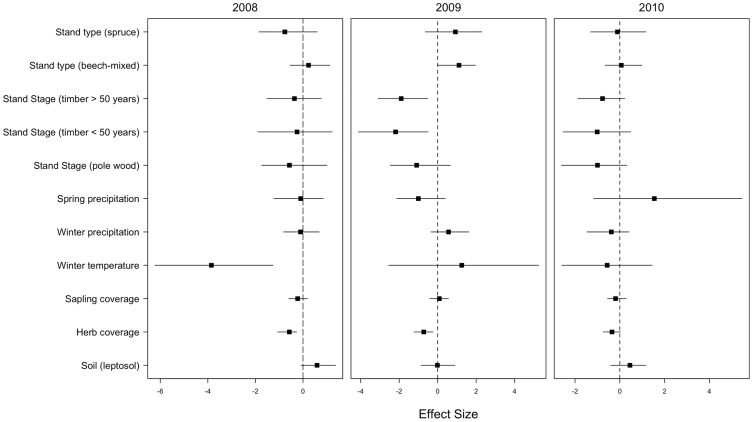
Effect sizes of environmental covariates on nymph densities in each of the three sampling years 2008–2010 as estimated by single-year models. Effect sizes are given as posterior modes and 95% highest posterior density estimates. The baseline for stand type is ‘beech’, for stand stage ‘thicket’ and for soil ‘cambisols’.

Single-year analysis of questing nymph density revealed the largest effect size for winter temperature in 2008 with fewer ticks at increasing temperature, explaining 88% of the variance in nymph activity. Notably, this effect contrasts with the winter temperature effect on nymphs from the full model. In the latter, the temperature effect is mainly driven by the pronounced overall yearly differences (Figure 3). In contrast, the effect size was largest for the spring precipitation in 2010, with more nymphs being sampled with increasing spring precipitation, accounting for 57% of the variation in nymph activity during 2010; in 2010, spring precipitation was considerably lower than in the two preceding years.

## Discussion

Our study has identified forest successional stage as an important predictor of nymph questing density of *Ixodes ricinus* in production forests in SW Germany. Nymph questing density was strongly linked to the preceding winter temperature, especially to overall yearly changes in weather conditions. The variable prediction strength of environmental covariates across years, the contrasting effect of winter temperature on nymphs from a single year, and the pronounced effects of weather across years suggest that more robust prediction can be achieved through long study periods only. We highlight that this study only examined the relative tick questing density in different forest stands, whereas the strong dependence of tick survival and reproduction on host accessibility makes it clear that tick density is not independent of host abundance and diversity from local to regional scale. Increasing deer abundances in Europe may have caused increases in tick populations recently, and both abiotic and biotic environmental factors are likely to have caused recent increases in tick populations [36].

Increased nymph densities in younger forest stands and the weak effect of stand type is surprising, as tick abundance has previously been suggested to be favored by canopy closure, which is higher in older forest stands. In contrast, a pronounced shrub and herbaceous layer, which was not present at our research sites in younger forest stands, and particular litter types have been suggested to favor *I. ricinus* abundance and survival elsewhere in Europe [11], [37], [38] and *I. scapularis* in North America [17], [39]. Whereas forest classification into stand composition and successional stages comprises rather coarse measures, this classification can be applied at a larger spatial scale through digital forest inventories and management maps. These are readily available for most managed forest stands in Central Europe and elsewhere (see also [11], [40]). Managed forests mostly consist of patches with homogeneous tree composition, age structure, and vertical organization. Simplification of the key characteristics might therefore help to predict the consequences for the occurrence and distribution of species on a larger scale and under future scenarios of forest development and global change. Other factors, such as herb and shrub coverage, might influence tick occurrence and survival by being associated with more suitable microhabitat [11], [38] but are more difficult to extrapolate to a larger spatial scale. Herb and shrub coverage are largely variable within and across different forest stands.

In contrast to our expectation that tick densities rise with increasing herb and shrub cover, we found the opposite trend for herb coverage. Fewer nymphs were associated with increasing herb cover. We have no explanation for this finding. Perhaps other factors linked with herb coverage, such as specific habitat preferences of rodent host species where herb coverage dominates, might have led to these results. Larger tick populations in young forest stands, for example, might be linked to an increased abundance of rodent hosts such as *Myodes glareolus* [41] or *Apodemus flavicollis* [42] in these forest. Wood mice *Apodemus sylvaticus* were found to aggregate under *Rhododendron* shrubs in southern England, but questing tick density was lower under these shrubs compared to surrounding forest habitat [43]. Variation in host abundance blur the possible impact of habitat features such as herb coverage or other environmental variables. Notably, in addition to the possible role of host densities on tick abundance and questing behavior, the tick burden of rodent hosts was also found to vary with relative humidity and vegetation cover at other study sites in Germany [44]. Moreover, sampling bias such as the less effective sampling of areas with high vegetation might well influence drag sampling results [29].

Our study results are preliminary in that our coarse sampling procedure provides only a first estimate of relative tick questing densities. The number of drag-sampled tick individuals cannot be used to distinguish local tick density and their questing behavior, as both factors determine the number of ticks that are counted. Further, we have no information on important factors such as the abundance of potential host species. The sampling period of three years does indeed highlight that the strength of environmental factors on tick density varies over years, but possible interactions and the covariation of habitat, weather, and host species are hardly detectable from such short-term studies.

Nevertheless, we expect our finding of increased questing nymph densities in young forest stands to be important as an indication that forest management practices have an impact on tick occurrence and therefore also possibly on disease transmission risk on the stand level. In practice, the large number of abiotic and biotic factors that could influence tick questing density suggests that systematic sampling with an accurate sampling procedure and replicates over a time-scale of years is needed. Such a sampling method will directly link local tick abundance to various potential environmental forces. Integrative monitoring efforts ideally would also include surveys that allow accurate abundance estimates of various vertebrate host species, such as rodents and deer [43], [45].

This is particularly important, since to date, the environmental factors suggested to influence tick population dynamics are ranked inconsistently in their importance [46], [47]. The combination of diverse aspects in a single analysis is also a prerequisite for distinguishing various kinds of direct and indirect relationships typically shaping interactions in disparate environments [48], [49]. Only when the different sources of variation are determined, can we understand the genuine functional relationships between interacting organisms and various environmental attributes, which in turn ultimately determine questing tick density in time and space. Variability in the relative importance of environmental forces during the course of our three-year sample study is one major obstacle to disentangling the influence of even a limited number of environmental forces on tick density. We have observed, for example, a significant increase in questing nymph density after the mild winter in 2008. Milder winter temperatures have also been identified as important aspects for the northward expansion of *I. ricinus* [50]. Our short sampling period of three years however urges caution in concluding that a direct relationship exists between winter temperature and tick density. Notably, the increasing winter temperature across sites in 2008 resulted in fewer nymphs in contrast to the increased tick densities associated with the milder yearly winter temperature. This demonstrates that conclusions from single-year studies might differ from long-term studies [9], and moreover, that the effects of environmental forces might differ in spatiotemporal dimensions.

This finding and, in particular, our conclusion that milder weather conditions favor higher tick densities need further research. Such aspects in general have rarely been adequately examined with sufficiently large samples [39]. Notably, the gridded weather data extrapolated from regional weather stations comprise so far only a coarse and preliminary measure. These coarse-grained data might therefore not fully account for actual gradients in microclimatic conditions. Moreover, microclimates in forests are well known to be influenced by local stand attributes [51], [52], and the true gradient in microclimatic conditions might therefore be even more pronounced. Tick questing behavior and activity is strongly linked to temperature and humidity [53]. Pronounced yearly weather changes might therefore also alter the relative effect of habitat features over periods of years. During heat waves in the summer, for example, beech forests have been found to have a more moderating effect on maximum daytime temperature than spruce forests [54]. Spruce forests are relatively warmer during minimum temperatures as a consequence of the different amounts of radiation reaching the ground. In young forest stands of thicket and pole wood, which lack pronounced canopy layers, the level of radiation reaching the ground is high and fosters a rise in soil and air temperatures and a decrease in ground humidity during hot weather.

We suggest that the possible variation in the effects of environmental factors on tick density over years needs to be addressed in future research, and that possible covariances in the effects of environmental factors should be considered. If the effective weather and microclimatic conditions differ across habitat types as discussed above, a likely (but to our knowledge poorly investigated) consequence is that the relative effects of habitat features such as tree composition and forest stage will differ with the varying weather conditions. Given the technical limits of accurately fitting the interactions and dynamics of population fluctuations from two to three years of data, as is common for many tick studies, the lack of meaningful random effects in our study is not a surprise. In addition to the need for the monitoring with sufficiently replicated sampling in time and space, field studies of tick abundance and distribution would benefit from research frameworks that assess a suite of relevant and standardized abiotic and biotic covariates to improve the disentanglement of the relative strength and possible relationships between the various factors recorded as influencing tick occurrence to date.
